# Interleaving Automatic Segmentation and Expert Opinion for Retinal Conditions

**DOI:** 10.3390/diagnostics12010022

**Published:** 2021-12-23

**Authors:** Sergiu Bilc, Adrian Groza, George Muntean, Simona Delia Nicoara

**Affiliations:** 1 Department of Computer Science, Technical University of Cluj-Napoca, 400114 Cluj-Napoca, Romania; sergiubilc90@gmail.com; 2 Department of Ophthalmology, “Iuliu Hatieganu” University of Medicine and Pharmacy, Emergency County Hospital, 400337 Cluj-Napoca, Romania; georgemuntean99@gmail.com (G.M.); simonanicoara1@gmail.com (S.D.N.)

**Keywords:** optical coherence tomography, retina layer segmentation, geodesic distance, vertical and horizontal gradients, human-centered AI

## Abstract

Optical coherence tomography (OCT) has become the leading diagnostic tool in modern ophthalmology. We are interested here in developing a support tool for the segmentation of retina layers. The proposed method relies on graph theory and geodesic distance. As each retina layer is characterised by different features, the proposed method interleaves various gradients during detection, such as horizontal and vertical gradients or open-closed gradients. The method was tested on a dataset of 750 OCT B-Scan Spectralis provided by the Ophthalmology Department of the County Emergency Hospital Cluj-Napoca. The method has smaller signed error on layers B1, B7 and B8, with the highest value of 0.43 pixels. The average value of signed error on all layers is −1.99 ± 1.14 px. The average value for mean absolute error is 2.60 ± 0.95 px. Since the target is a support tool for the human agent, the ophthalmologist can intervene after each automatic step. Human intervention includes validation or fine tuning of the automatic segmentation. In line with design criteria advocated by explainable artificial intelligence (XAI) and human-centered AI, this approach gives more control and transparency as well as more of a global perspective on the segmentation process.

## 1. Introduction

Optical coherence tomography is a non-invasive imaging method that revolutionized ophthalmology and whose continuous improvement opened new perspectives on the management of retinal diseases. However, the high number of people with retinal conditions requiring constant OCT monitoring results in a significant burden for ophthalmologists. Simultaneously, the evolution of artificial intelligence (AI) has led to the development of methods capable of performing high-level analysis of images and other data. Consequently, AI-based healthcare services are expanding, with expectations of USD 6.6 bn by the end of 2021.

Optical coherence tomography (OCT) has become the leading diagnostic tool in modern ophthalmology [1,2,3], providing various OCT-based biomarkers, including retinal thickness, intraretinal cystoid fluid, subretinal fluid, alterations of outer retinal layers, and hyperreflective foci.

While interpreting the OCT, the ophthalmologist uses both qualitative and quantitative features contained in the OCT images. The first evaluation concerns the overall aspect of the macula on the OCT B-scan corresponding to the fovea. Global retinal deformations, such as concavities or convexities, can be discovered. At this point, a thorough examination of the retinal zones’ integrity is conducted. The same elements should be checked for the rest of the acquired B-scans. The next step would be looking at the retinal thickness (adjusted for age and gender) corresponding to the nine zones contained in the Early Treatment Diabetic Retinopathy Study (ETDRS) macular grid. Increases or decreases could indicate pathological changes.

While measuring the total retinal thickness using automatically generated thickness maps provided by the OCT gives us a good overall impression over the health of the retinal structures, measuring each layer separately has its own advantages.

It can help us see which layer is affected, thus pointing towards a possible diagnosis. The retinal nerve fiber layer (RNFL) and ganglion cell layer suffer in glaucoma and multiple sclerosis, the inner retinal layers can become thinner after retinal artery occlusions, the inner nuclear layer can become thicker after microcystic macular edema, portraying evidence of CNS inflammation, and the outer retinal layers show signs of alterations in conditions affecting the retinal pigment epithiulium (RPE) like age-related macular degeneration (AMD) or central serous corioretinopathy (CSCR).

Looking solely at the overall thickness, we might overlook changes, such as in the case of drusen—the hallmark of the age-related macular degeneration non-exudative type. While drusen increase, the outer retinal structures (outer nuclear layer, photoreceptor layer and retinal pigment epithelium) get thinner in a manner that is proportional to the drusen; hence, the total thickness remains unchanged. Besides showing increased thickness, automated segmentation may show alterations of the profile of these layers.

The automatic segmentation of retinal layers can play a role in computer-aided classification. While training a classifier to distinguish between macular changes associated with age-related macular degeneration and those with diabetic macular edema, we can choose certain layers as boundaries. Thickness increases beneath or above this layer can help strengthens the classifiers’ accuracy.

Finally, advances in segmentation can help diagnose incipient changes in the course of retinal conditions.

The standard software of OCT devices includes algorithms for the automatic segmentation of layers, which are, e.g., based on: analysis of the image brightness, the technique of active contours, pattern recognition, graph theory, or techniques of grouping. Automatic layer segmentation of the retina in the OCT images requires overcoming many challenges, such as noise, an uneven reflection of light by the tissues, absorption of light through the blood vessels, an unexpected movement of the patient, and the dependence of the proper segmentation algorithm on the device. Particular difficulties are also introduced due to the presence of lesions.

Our approach relies on graph theory and geodesic distance. Since each retina layer is characterised by different features, we interleave various gradients during detection, such as horizontal and vertical gradients or open-closed gradients. Our target is a support tool for the human agent. Hence, we focus on allowing the user to intervene after each automatic step. Human intervention includes validation or fine tuning of the automatic segmentation. In line with design criteria advocated by explainable AI (XAI), this approach gives more control and transparency as well as more of a global perspective on the segmentation process.

## 2. Diagnosing Incipient Changes

Diabetic macular edema (DME) is the primary reason for vision loss associated with diabetic retinopathy (DR). It is generated by abnormal vascular permeability. The diagnosis is made through binocular slit-lamp biomicroscopy, identification of leakage on fluorescein angiograms (FA) or by examining the retinal structures and thickness provided by OCT. Compared to OCT, the first two methods are deemed to be subjective and may overlook small changes in retinal thickness [4]. Increased thickness of the macular region for a patient with DR usually indicates DME, and this can be quantified by measuring the entire retinal thickness with OCT-generated maps.

Advances in retinal layer segmentation has shown changes less obvious that seem to precede increased macular thickness. For example, patients with diabetes without DR or with initial DR show decreased retinal nerve fiber layer (RNFL) and increased inner nuclear layer (INL)/outer plexiform layer (OPL) thickness versus controls [5]; similarly, patients with type 2 diabetes with minimal DR show thinner inner retinal layers of the macula compared with healthy controls [6].

Measuring each layer separately can help us see more subtle changes and implement the necessary treatment faster.

Age-related macular degeneration (AMD) is one of the most common causes of vision loss worldwide. In Europe, in 2040, the number of individuals with early AMD is expected to range between 14.9 and 21.5 million, and the number of individuals with late AMD is expected to range between 3.9 and 4.8 million. Low vision severely impacts the quality of life among populations and poses an enormous global financial burden.

Increased thickness of the retina (after adjusting for age and gender) usually indicates changes that come with the exudative type. Following the growth of the new vessels from the underlying choroid (type 1 or type 2) or from the retinal deep capillary plexus (type 3), the retinal architecture is changed and retinal thickness increases. The changes account for the growth of the vessels, the fluid that leaks from the weak vessel walls and the fibrovascular scar that is formed over time.

Decreased thickness of the retina (after adjusting for age and gender) appears in the late stages of the non-exudative type, with the loss of RPE, the internal segment ellipsoid and the photoreceptor layer. The changes are named non-geographic atrophy or geographic atrophy depending on whether the loss is localized or if it covers a contiguous area.

The before-mentioned changes that increase or decrease the overall thickness of the retina occur in the last stage of the disease when the visual impairment has already settled. More fine changes appear earlier in the course of the disease, but these may become clear only after segmentation and measurement of the individual retinal layers. When comparing patients with no AMD to those with moderate and severe early AMD, we can see changes in retinal layer thickness: in the latter, the RPE/Bruch’s membrane (BM) complex thickness is increased, while the photoreceptor inner/outer segments, interdigitation zone and outer nuclear layer (ONL) thickness are decreased [7].

Catching these early changes can help us more closely monitor these patients and could play a role in observing the response to the newly developed treatments in various clinical trials.

## 3. Segmentation of Retinal Layers

We build here on the work of Duan et al. [8], whose method for OCT image segmentation is based on graph theory and geodesic distance. The geodesic distance between two vertices is the number of edges of the shortest path between the vertices. In our case, the geodesic distance is computed based on an exponential function weighted by the gradient.

Differently from most segmentation methods, we use here both the vertical and horizontal gradient. The rationale is to improve segmentation, especially in cases with retina conditions. In case of retina conditions, the layers may have a weaker contour, the curves are larger (e.g., caused by inter-retinal fluid), or the layers can even be interrupted (e.g., caused by drusens).

**Figure 1:**
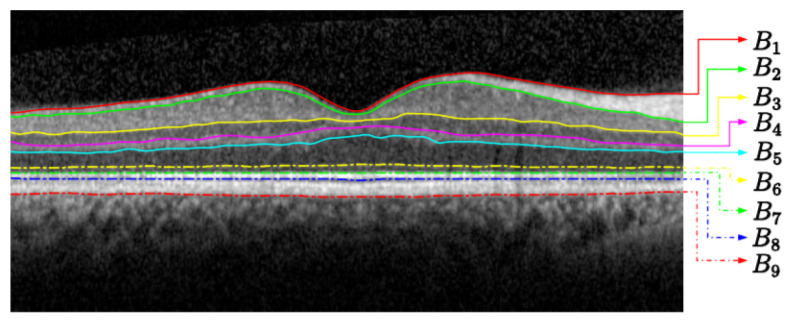
Retina segmentation from an OCT B-Scan image.

Our segmentation strategy is to firstly detect the most visible layer, the seventh layer IS-OS (see Table 1). Then, we determine a region of interest for each of the remaining eight layers. Each region is relative to the IS-OS layer firstly detected. We subsequently compute the geodesic distance within each region by following two steps. First, we compute the weighted geodesic distance (WGD) by employing a fast sweeping method [9]. Second, we use the WGD value to determine the layers by solving a differential equation using a descendant gradient. These steps are used to detect the nine layers in the retina (see Figure 1). By splitting the image into eight areas of interest, we avoid detecting the same most visible layer (i.e., IS-OS or ILM; recall Table 1).

### 3.1. Detecting the IS-OS Layer

To detect the IS-OS layer (i.e., B7), we use a local algorithm for adaptive binarisation. The algorithm is based on the formula:

(1) $$p=\left\{\begin{array}{cc} 0, & l_{s}\left(I,ws\right)-I>C\\ 1, & otherwise \end{array}\right.$$

where *I* is the given OCT image and $l_{s}\left(I,ws\right)$ is the image after a median filter of size ws is applied to *I*. The filter aims to prepare the image for binarisation by applying the Split–Bregman algorithm [10]. The parameter *C* controls the increase of image variations based on which binarisation takes place. We used a median filter of size ws=100, while C=0.01.

The Algorithm 1 formalises the method for computing the IS-OS frontier of the input OCT image *I*. The output of the Compute7THLayer function is a vector of 512 elements, 1 for each column of the given OCT image. Each value represents the vertical position of the frontier in the corresponding column. If the detection is not accurate enough, a human agent can intervene by fine tuning the coefficients of the algorithm.
**Algorithm 1.** Computing the IS-OS frontier.1:**function** compute7thLayer(I,pars)2:    $bwImg=Seg_{2}\left(I,100,pars.Enh,pars.Smooth\right)$3:    img=bwImg⨂image4:    gradImg=getGradientMap(img,5:                         darkToBright,params)6:    [B7,Y]=getSegBoundary(gradImg)7:    return[B7,Y]8:**end function**

### 3.2. Double-Checking the Segmentation of the IS-OS Layer

**Figure 2:**
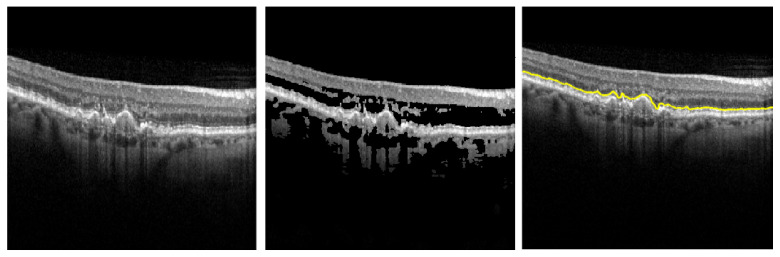
Modifying the initial OCT image (**left**) with an adaptive thresholding algorithm based on Equation (1) (**center**) aiming to accurately detect the easiest frontier between the IS and OS layers (**right**).

**Figure 3:**
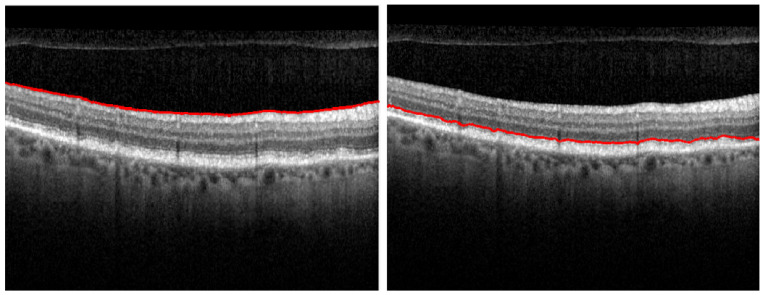
Example of the segmentation of the IS-OS boundary before and after the correction of the segmenting parameters.

Since identifying the frontier between IS and OS layers is the key element in our approach (as the other layers depend on it), we need to assure its correctness. Our assumption is that following this adaptive thresholding algorithm, the highest intensity variation is on the IS-OS layer. Figure 2 bears this out. The system displays to the ophthalmologist only this layer (i.e., B7), in order for the human agent to check the validity of the segmentation (see Figure 3).

### 3.3. Detecting the Remaining Layers

Detecting the rest of eight layers is relative to the identified frontier between IS-OS (i.e., B7). The B7 layer is used to set two areas of interest around it. The benefit of such areas of interest is that the method for computing geodesic distance avoids being kept in local maxima. The layers below IS-OS (i.e., those on the external part of the retina) are detected as follows.

We continue by considering the RPE-CH (i.e., B9), which is the lowest retinal layer. To detect the B9 layer, we rely on the algorithm proposed by Duan [8], in which the weighted function W(x) is given by:

(2) $$W\left(x\right)=\left\{\begin{array}{c} 1-\exp(-\lambda \left(1-n\left(\nabla_{x}I\right)\right)n(|\nabla_{y}I|)),\;\;c-o\\ \exp(-\lambda \left(1-n\left(\nabla_{x}I\right)\right)n(|\nabla_{y}I|)),\;\;\;\;\;o-c \end{array}\right.$$

Here, *I* is the current OCT image, *n* is a linear operator used for normalisation, and λ is a user-defined parameter that gives that intensity level from the area of interest. Next, $\nabla_{x}$ and $\nabla_{y}$ are first-order gradient operators on the *X* and *Y* axes. $n(|\nabla_{x}I|)$ is the positive horizontal gradient used to detect intensity variations on the horizontal direction, occurring often when the retina suffers from a certain condition.

We also consider the type of color difference between each layer. For instance, when segmenting a layer that absorbs the light (that is darker), the intensity variations are more visible in the magnitude of the open-close gradient (i.e., noted with o-c in Equation (2)). Differently, when finding the frontier between a darker layer and a brighter one, we use the close-open gradient (i.e., noted with o-c in Equation (2)). Since we are interested in the RPE-CH (B9), we apply the open-close gradient on the area containing pixels below the frontier between IS and OS.

Then, the remaining layers are computed as follows:

1 OS-RPE (B8) uses the open-close gradient in the area between B7 si B9;
2 ONL-IS (B6) is the frontier situated above the B7 layer. We use an area of interest limited by B7 and a parallel line situated at a distance that can be adjusted by a human expert;
3 ILM (B1) is a frontier with high variation of intensity, and hence, is more easily detectable. To increase the accuracy, the B7 layer is also considered during the detection of B1;
4 INL-OPL (B4) uses the close-open gradient applied to the region between ILM and ONL-IS;
5 OPL-ONL (B5) is detected in the area situated between B4 and B6 by means of the close-open gradient;
6 IPL-INL (B3) is detected in an area situated above B4 and below a parallel line of some 20 pixels. The open-closed gradient is applied.

### 3.4. Checking the Areas of Interest

Since we target a support tool for the human agent, we allow the user to intervene after each automatic step. The human intervention includes validation or fine tuning of the automatic segmentation. This approach gives more control and transparency as well as more global views of the segmentation process. Note that no black box machine learning has been used.

**Figure 4:**
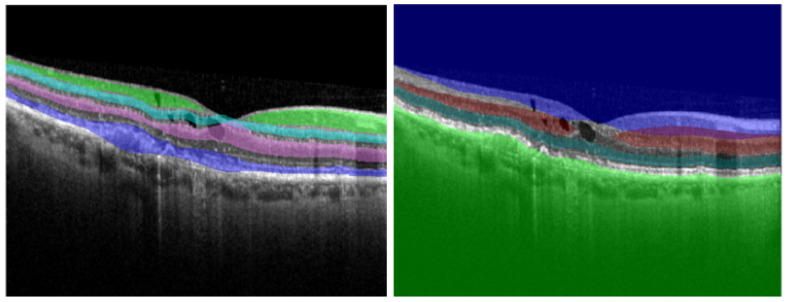
Computing the areas of interest for each layer: B2, B4, B6 and B7 (**left**) and B1, B3, B5 and B8 (**right**).

After detecting the IS-OS layer, it is important to limit the search for boundaries for the remaining layers to only be in the areas of interest for each specific boundary. Therefore, a human agent is able to preview the regions of interest of these new frontiers, which are sequentially calculated based on the previously segmented layers (see Figure 4). Then, the human-in-control can assess if the regions are correctly overlapping the layer which needs to be segmented.

Various interactive features have been developed to present to the human agent the regions of interest of each frontier and to allow him to interact with the segmentation algorithm. In this line, the user can change the thickness of a region of interest and also the distance from that region to the nearest neighboring frontiers. An example of such an interactive function appears in Algorithm 2. Since we are interested here in determining the frontier B2 (RNFL), we compute the region of interest with respect to B1 and B3. The functions *removeRegionAboveSegLine(B,Y,img,bandwidth)* and *removeRegionBelowSegLine(B,Y,img,bandwidth)* are used to remove the area with the thickness of bandwidth pixels situated under, respectively above, the frontier *B*. The benefit is that the parameters within the structure par can be modified by the human agent from the tool’s interface. The outputs of these functions represent binary masks with the same size with the given OCT image. Once computed, each binary mask is added to a data structure that is used to flexibly display each region of interest to the human agent.
**Algorithm 2.** RNFL segmentation by firstly computing its region of interest.1:**function** RNFL(gradImg,B1,par)2:    BW1=rmvRegAboveLine(B1,par.AbvWidth)3:    BW0=rmvRegBelowLine(B1,par.BlwWidth)4:    BW=BW0⨂BW15:    gradImg=getGradientMap(imgPad,brightToDark)6:    ROI=gradImg⨂BW7:    [B2,Y]=getSegBoundary(ROI)8:    return[B2,BW]9:**end function**

**Figure 5:**
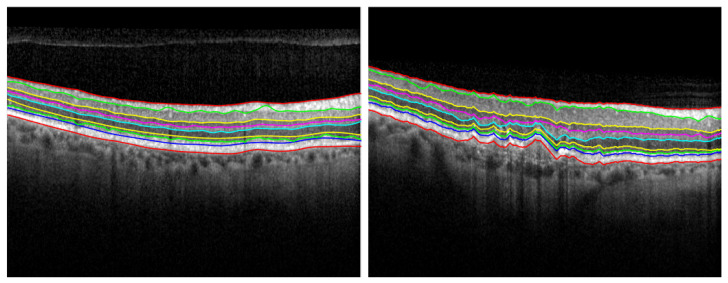
Retinal segmentation on a healthy retina on the left side, while segmentation of a pathological retina is presented on the right.

If the areas of interest do not match the corresponding layer, the human agent can modify the parameters and call the function again on the adjusted parameters. The frontiers are obtained with the function getSegBoundary applied on the image computed by multiplying the gradient map with the binary mask of the area of interest of the current edge. The results of the algorithm are illustrated in Figure 5.

## 4. Exemplifying Frontier Detection

Next, we exemplify the process of segmenting a single layer, since the method of extracting the boundaries is similar for the remaining layers.

The first step in segmenting the retinal layers in our solution is detecting the IS-OS boundary. To ensure that B7 will be the most prominent boundary, we enhance the pixels around the specified layer using a method described by Algorithm 3. The idea behind this method is to apply a mean filter with a high window size on the original image and to subtract the image from the result of the filter. The output of this subtraction will be a matrix with the same size as the image containing negative values in the pixels which were originally below the average intensity. Those pixels are then ignored by computing a binary mask by applying a threshold of 0.
**Algorithm 3.** Enhacing the IS-OS layer.1:**function** Seg2(IM,ws,enh,smooth)2:    IM=denoise(IM,smooth)3:    mIM=average−filter(IM,ws)4:    sIM=mIM−IM−enh5:    bw=threshold(sIM,0);6:    bw=complement(bw)7:    return[bw]8:**end function**

After applying the binary mask on the original image, thus enhancing it and ensuring the fact that IS-OS will be the first segmented boundary, the next step is to apply Algorithm 2 on the enhanced image. This algorithm consists of three steps.

First, we compute the region of interest for the current boundary. Each region of interest needs to be narrowed based on their adjacent layers to avoid reaching a local maxima problem.

Second, we compute the gradient of the image. Algorithm 4 applies Equation (2) to denoise the image using the SplitBregmanROF algorithm. Then, the algorithm calculates and normalizes both gradients and applies them in the exponential function from the Equation (2).
**Algorithm 4.** Computing the gradient of the OCT image.1:**function** getGradientMap(img,flag,ws,smooth)2:    IM=denoise(IM,ws,smooth)3:    [gradImgX,gradImgY]=gradient(IM)4:    **if** flag==0 **then**5:        gradImgY=−gradImgY6:    **end if**7:    gradImgY=normalise(gradImgY,[0,1])8:    gradImgX=abs(gradImgX)9:    gradImgX=normalise(gradImgX,[0,1])10:  gradImg=1−(1−gradImgY)⨂gradImgX11:  gradImg=normalise(gradImg,[0,1])12:  return[gradImg]13:**end function**

The function *getSegBoundary(gradImg)* presented in Algorithm 5 is used to segment the current boundary based on the gradient map previously calculated. In this example, the function consists of transforming the digital image into a weighted graph and applying Dijkstra’s shortest path algorithm to obtain the desired boundary. The same result can be reached using a fast sweeping approach.
**Algorithm 5.** Computing layers boundaries by extracting the most prominent boundary available.1:**function** getSegBoundary(gradImg)2:    adjMatrix=getAdjMatrix(gradImg)3:    [X,Y]=Dijkstra(adjMatrix)4:    return[X,Y]5:**end function**

Once the IS-OS is extracted, the other boundaries can be segmented using the same process. The only difference is that the function getSegBoundary is now applied only on the region of interest of the current layer.

## 5. Running Experiments

### 5.1. Dataset Description

First, we used a dataset of 750 OCT B-Scan Spectralis obtained from the Ophthalmology Department of the County Emergency Hospital Cluj-Napoca. Second, we used the dataset of Chiu et al. [11] to compare our method with the existing one. The Chiu dataset contains images from both healthy retinas and retinas with conditions. Each image was manually segmented by two specialists. Third, we used a 40-image dataset to validate frontier extraction. Since Chiu et al. detect only eight layers, to compare the results, we did not consider the segmentation for ONL-IS (i.e., B6).

### 5.2. Evaluation Metrics

We used both signed error (SE) with SE $\left(B_{i},B_{i}^{\prime }\right)=\frac{1}{n}\sum_{j=1}^{n}\left(B_{ij}-B_{ij}^{\prime }\right)$ and the mean absolute error given by MAE $\left(B_{i},B_{i}^{\prime }\right)=\frac{1}{n}\sum_{j=1}^{n}\left(\right|B_{ij}-B_{ij}^{\prime }\left|\right)$. Here $B_{i}$ represents the frontiers detected by automatic segmentation, while $B_{i}^{\prime }$ are the manually annotated frontiers. A smaller SE indicates smaller differences between frontiers. The signs of SE indicates if the detected frontier is above or below the true frontier.

**Figure 6:**
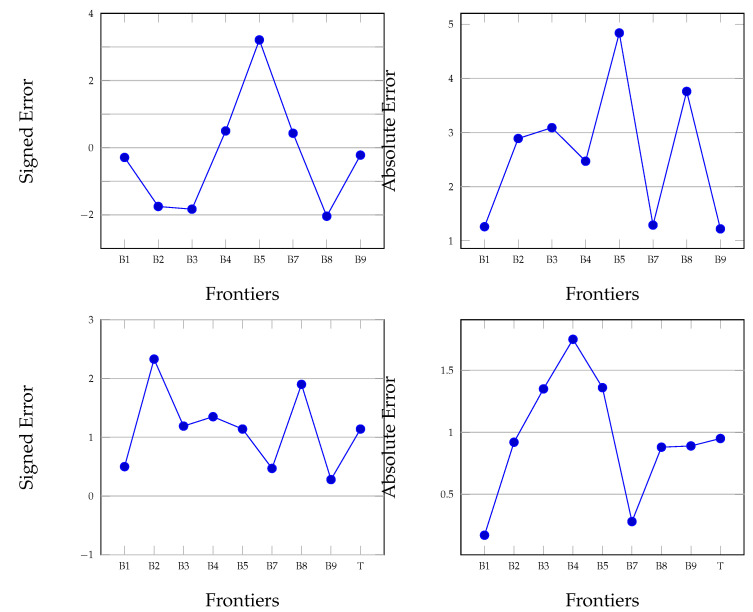
Mean (**top**) and standard deviations (**bottom**) for SE (signed error) and AE (absolute error) for healthy retinas.

We analysed the segmentation results with distinct OCT-sets: one containing normal retinas, and the other retinas with conditions. For the normal retina, the mean and standard deviation are listed in Table 2. Our method has smaller SE errors on layers B1, B7 and B8. The highest value is 0.43 pixels. SE has values around 2 px for the layers B2, B3, B5’ and B8. The standard deviation is similarly distributed with maximum values of 0.5 pixels for the layers B1, B7 and B9, respectively, and 1.52 px for B2, B3, B5 and B8. The average value of SE on all layers is −1.99±1.14 px. The average value for AE is 2.60±0.95 px. These results are also depicted in Figure 6. To compare our method with the one proposed by Chiu et al., we list here their values for SE (−1.20±0.54 px) and AE (2.09±0.59 px).

**Figure 7:**
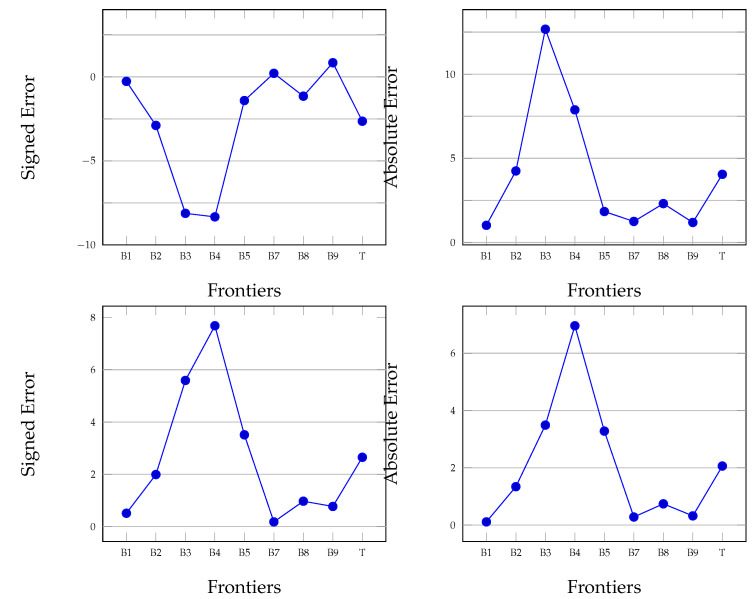
Mean (**top**) and standard deviations (**bottom**) for SE (signed error) and AE (absolute error) for retinas with conditions.

In the case of retinas with conditions, the results are depicted in Table 3 and Figure 7. One observation is that errors are larger in the case of abnormal retinas. The frontiers B3 and B4 have larger errors, with values above 8 px. This result is given by the fact that in the case of retinal conditions such as DME, the fluid is accumulated in these layers.

One advantage is that the mean error ME is small: its value is less than 1 px for B1, B7 and B9. This low error is important for a correct segmentation of the retina. The same case is with the absolute error: The frontiers B2–B4 have errors between 4.25 and 12.57 px, while the remaining frontiers have segmentation errors within 1−2 px. The mean value for ME is −2.64±2.65, while for AE this value is 4.05±2.06 (the tool is available at https://github.com/BilcSergiu/OCT-Segmentation, accessed on 16 November 2021).

**Figure 8:**
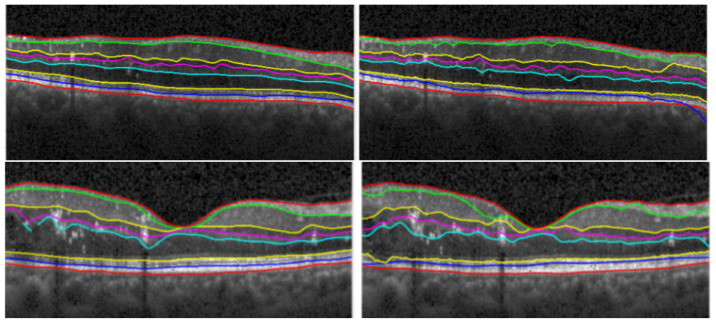
Comparing the results of manual segmentation (**left**) to automatic segmentation (**right**) on a healthy retina (**top**) and on a retina with condition (**bottom**).

Even though the OCT B-scan’s accuracy of correctly representing the individual retinal layers is almost comparable to in vivo histopathology (Figure 8), manually delineating these layers is a time consuming task that can’t be fitted in a regular clinical practice routine.

## 6. Discussion and Related Work

Dufour et al. have proposed in [12] a segmentation method of OCT retina images based on graphs. This method takes into consideration weak constraints or knowledge obtained from previous segmentations, and it is based on energy minimisation. The performance is higher in the case of healthy retinas compared with those patients presenting drusen within the retina’s layers. The mean of the absolute error was 3.05±0.54μm. The approach of Dufour et al. targeted both healthy retinas and those with macular edema, but focused on detecting only six frontiers: ILM, RNFL, IPL-INL, IS-OS, and RPE-CH.

Chiu et al. [11] have detected retina layers from OCT images by computing the minimum distance between two points from the graph G=(V,E), where *V* is the set of pixels from the B-SCAN and *E* is the set of weights assigned to each pair of nodes from the graph. Each pixel from the initial image is connected to its eight neighbours. The weights for two nodes are given by the intensity variation of the image on the vertical axis. The variation is given by the intensity of the vertical gradient. Based on these values, the frontier with the highest intensity variation is identified. This is the same as finding the minimum cost distance (i.e., rDijkstra algorithm) between the first and the last node in the set *V*. The method developed by Chiu et al. detects seven frontiers: ILM, RNFL, IPL-INL, INL-OPL, OPL-ONL, and IS-OS. The mean error of thickness of each layer between automatic segmentation and human expert segmentation was 0.9±0.82.

The Caserel project [13] aims at assisting the segmentation of retina layers from OCT images. Similar to Chiu et al. [11], the segmentation method in Caserel uses graph theory, the minimum cost algorithm, and gradients. Each layer is successively identified based on the previous one until the following six frontiers are obtained: ILM, NFL-GCL, IPL-INL, INL-OPL, OPL-ONL, IS-OS, and RPE.

Duan et al. have also exploited the notion of geodesic distance [8]. The geodesic distance is computed by using an exponential function based on both vertical and horizontal gradients. During computation a fast sweeping algorithm is used. Similar to Chiu et al., the method relies on the maximum difference in intensity between two layers, which makes both methods sensible to local maximum. In a method different than that employed by Chiu et al., Duan et al. have used a different function to compute the weights between two nodes. The method is able to segment nine layers in the retina: ILM, RNFL, IPL-INL, INL-OPL, OPL-ONL, ONL-IS, IS-OS, OS-RPE and RPE-CH.

We tackle here the task of segmenting 2D OCT images, while some consideration has been recently given to improving the segmentation of 3D retina layers. One such example is the work of Stankiewicz, which applies graph theory to deal with low quality OCT images [14].

Compared with the above approaches, the main difference is that we focus on the interaction with the user. Since we target a support tool for the human agent, we allow users to intervene after each automatic step. The human intervention includes validation or fine tuning of the automatic segmentation. In line with the design criteria advocated by explainable AI (XAI), this approach gives more control and transparency as well as a more global perspective on the segmentation process. Note also that no black-box machine learning has been used. Therefore, in the context of a plethora of deep-learning segmentation approaches [15], we consider this interactivity with the human expert as a distinctive feature.

In line with the Explainable AI principle, we analyse when the proposed solution is not reliable. As far as we see, the utility of segmentation ranges from catching early incipient changes and guiding the clinician in the direction of a certain diagnosis to helping in the preprocessing step of a computer-aided classification or prediction task.

One of the limitations of our method is related to the patient’s retinal condition. The intervention of the ophthalmologist might be necessary when the retinal structures undergo important alterations due to the advanced stages of certain disorders or when vital layers in the segmentation process suffer modifications. First, if a significant volume of retinal fluid infiltrates between the retinal layers, our solution will have problems detecting the IS-OS boundary, therefore affecting the segmentation of the remaining boundaries. These changes can appear in advanced cases of diabetic macular edema or age-related macular degeneration. Second, if changes related to the presence of drusen or fibrovascular scars appear around the RPE layer, our solution will not be able to properly segment the OS-RPE and RPE-CH boundaries because the segmentation is currently done in relation to the IS-OS boundary. These changes take place mainly in age-related macular degeneration, but they materialize in different stages of the disease. A final limitation where the human intervention correction could improve the automatic segmentation would be in the case of a poor signal due to media opacities, where the automatic segmentation might fail due to lack of clarity of the retinal layers.

In order to offer support for these limitations, the manual segmentation/correction of the preliminary results was added. A human agent can intervene and correct the results, both when the patient presents a high volume of fluid and when the patient presents drusen and fibrovascular scars.

## 7. Conclusions

We developed here a support tool for the segmentation of retina layers. The proposed method relies on graph theory and geodesic distance. As each retina layer is characterised by different features, we interleave various gradients during detection, such as horizontal and vertical gradients or open-closed gradients. The method was tested against two datasets which were publicly available and a third dataset of 750 OCT B-Scan Spectralis provided by the Ophthalmology Department of the County Emergency Hospital Cluj-Napoca. The method has smaller signed error on layers B1, B7 and B8, with the highest value of 0.43 pixels. The average value of the signed error on all layers is −1.99 ± 1.14 px. Average value for mean absolute error is 2.60 ± 0.95 px.

Different from the end-to-end machine learning approaches, the proposed system allows the human expert to intervene after each automatic step. Human intervention includes validation or fine tuning of the automatic segmentation. In line with design criteria advocated by explainable AI (XAI), this approach gives more control and transparency as well as a more global perspective on the segmentation process. In the context of many black box segmentation systems available, we consider this interactivity with the human expert as the main selling point of the system.

We are currently working on extracting knowledge from cases in which the human agent adjusts the parameters of our segmentation method. We plan to use such knowledge for feeding our algorithms with the aim of better handling complicated cases of OCT images.

## Figures and Tables

**Table 1:** Nine layers in the retina.

#	Layer Name	Abreviation
B1	Internal limiting membrane	ILM
B2	Outer boundary of the retinal nerve fibre layer	RNFL
B3	Inner plexiform layer-inner nuclear layer	IPL-INL
B4	Inner nuclear layer-outer plexiform layer	INL-OPL
B5	Outer plexiform layer-outer nuclear layer	OPL-ONL
B6	Outer nuclear layer-inner segments of photoreceptors	ONL-IS
B7	Inner segments of photoreceptors-outer segments of photoreceptors	IS-OS
B8	Outer segments of of photoreceptors-retinal pigment epithelium	OS-RPE
B9	Retinal pigment epithelium-choroid	RPE-CH

**Table 2:** Assessing the segmentation of healthy retinas.

Boundary	Signed Error (SE)		Absolute Error (AE)	
	μ	$\sigma^{2}$	μ	$\sigma^{2}$
ILM (B1)	−0.29	0.50	1.26	0.17
RNFL (B2)	−1.75	2.33	2.89	0.92
IPL-INL (B3)	−1.83	1.19	3.09	1.35
INL-OPL (B4)	0.50	1.35	2.47	1.75
OPL-ONL (B5)	3.21	1.14	4.84	1.36
IS-OS (B7)	0.43	0.47	1.29	0.28
OS-RPE (B8)	−2.04	1.90	3.76	0.88
RPE-CH (B9)	−0.22	0.28	1.22	0.89
Total	−1.99	1.14	2.60	0.95

**Table 3:** Segmentation performance in the case of retinas with conditions.

Frontier	Signed Error (SE)		Absolute Error (AE)	
	Mean	Std. Dev.	Mean	Std. Dev.
ILM (B1)	−0.27	0.51	1.02	0.11
RNFL (B2)	−2.89	1.99	4.25	1.34
IPL-INL (B3)	−8.12	5.59	12.67	3.49
INL-OPL (B4)	−8.33	7.68	7.88	6.96
OPL-ONL (B5)	−1.41	3.51	1.84	3.28
IS-OS (B7)	0.21	0.18	1.25	0.28
OS-RPE (B8)	−1.15	0.97	2.31	0.74
RPE-CH (B9)	0.84	0.77	1.19	0.32
Total	−2.64	2.65	4.05	2.06

## Data Availability

The data used to support the findings of this study are available on request from the corresponding author.
